# Capillary refill time for the management of acute circulatory failure: a survey among pediatric and adult intensivists

**DOI:** 10.1186/s12873-022-00681-x

**Published:** 2022-07-18

**Authors:** Matthias Jacquet-Lagrèze, Cléo Wiart, Rémi Schweizer, Léa Didier, Martin Ruste, Maxime Coutrot, Matthieu Legrand, Florent Baudin, Etienne Javouhey, François Dépret, Jean-Luc Fellahi

**Affiliations:** 1 Service d’anesthésie-réanimation, Hôpital cardiologique Louis Pradel, Hospices Civils de Lyon, 59, Boulevard Pinel, 69677 Bron Cedex, France; 2 Faculté de médecine Lyon Est, Université Claude Bernard Lyon 1, 8, Avenue Rockefeller, 69373, Lyon, Cedex 08 France; 3 CarMeN Laboratory, INSERM UMR 1060, University Claude Bernard Lyon 1, Lyon, France; 4 Service d’anesthésie-réanimation, Hôpital Louis Pradel, Hospices Civils de Lyon, 59, Boulevard Pinel, 69394 Lyon, Cedex France; 5 FHU PROMICE, DMU Parabol, Département d’anesthésie-réanimation, Hôpital Saint Louis, Assistance publique des Hôpitaux de Paris, Paris, France; 6 Faculté de médecine Paris, Université Paris France, Paris, France; 7 Department of Anesthesia & Perioperative Care, Division of Critical Care Medicine, University of California, San Francisco & F-CRIN-INI-CRCT Network, Nancy, France; 8 Service de Réanimation et Urgences Pédiatriques, Hôpital Femme Mère Enfant, Hospices Civils de Lyon, F-69500 Bron, France

**Keywords:** Capillary refill time, Acute circulatory failure, Survey, Peripheral perfusion, Shock

## Abstract

**Introduction:**

Recent studies have shown the prognostic value of capillary refill time (CRT) and suggested that resuscitation management guided by CRT may reduce morbidity and mortality in patients with septic shock. However, little is known about the current use of CRT in routine clinical practice. This study aimed to assess the modalities of CRT use among French adult and pediatric intensivists.

**Methods:**

A cross-sectional survey exploring CRT practices in acute circulatory failure was performed. The targeted population was French adult and pediatric intensivists (SFAR and GFRUP networks). An individual invitation letter including a survey of 32 questions was emailed twice. Descriptive and analytical statistics were performed.

**Results:**

Among the 6071 physicians who received the letter, 418 (7%) completed the survey. Among all respondents, 82% reported using CRT in routine clinical practice, mainly to diagnose acute circulatory failure, but 45% did not think CRT had any prognostic value. Perfusion goal-directed therapy based on CRT was viewed as likely to improve patient outcome by 37% of respondents. The measurement of CRT was not standardized as the use of a chronometer was rare (3%) and the average of multiple measurements rarely performed (46%). Compared to adult intensivists, pediatric intensivists used CRT more frequently (99% versus 76%) and were more confident in its diagnostic value and its ability to guide treatment.

**Conclusion:**

CRT measurement is widely used by intensivists in patients with acute circulatory failure but most often in a non-standardized way. This may lead to a misunderstanding of CRT reliability and clinical usefulness.

**Supplementary Information:**

The online version contains supplementary material available at 10.1186/s12873-022-00681-x.

## Introduction

Recent cohort studies have emphasized the prognostic value of capillary refill time (CRT) [1–3] and a recent clinical trial suggested that resuscitation management guided by CRT measurement may reduce morbidity and mortality in patients with septic shock [4, 5]. Subsequently, the clinical benefit has been endorsed by the recent guidelines of the surviving sepsis campaign [6, 7]. When used for research purposes, CRT requires a standardized approach encompassing the number of averaged CRT values, the level of pressure applied to the skin, the duration of skin compression, the localization of the test, and the use of a chronometer. Although CRT has proven to be useful and potentially lifesaving, little is known about its use in real-life conditions and how it is performed in routine clinical practice. It is however likely that CRT is both underused and misused and that healthcare providers do not strictly follow the standard at the bedside, creating a wide variability in practices. Such a sub-optimal use of CRT could lead to a misunderstanding of its reliability and clinical usefulness. To date, no study has focused on assessing the modalities of CRT use in routine clinical practice among adult and pediatric intensivists.

This cross-sectional survey aimed at describing and analyzing the practices in terms of CRT use among French adult and pediatric intensivists, in the setting of acute circulatory failure.

## Methods

The networks from the Société Française d’Anesthésie et de Réanimation (SFAR) and the Groupe Francophone de Réanimation et Urgences Pédiatriques (GFRUP) were solicited in September and October 2021 and asked to email, twice, an invitation letter for survey participation at each physician member of the networks, who are mainly adult/pediatric anesthesiologists and/or intensivists. The members of these learned societies are mainly from France, but also from Belgium, Algeria, Tunisia, Morocco, Luxembourg, and Canada. Responses of different physicians from the same institution were allowed. The invitation letter explained the purposes and benefits of the survey and gave a link to the dedicated web page (Survey Monkey, Palo Alto, CA, USA).

The survey included a total of 32 questions, XX of which were multiple choice questions, and is available in Additional files 1 and 2. The entire survey took approximately 10 min to complete. Two authors (CW and MJL) designed the questions and distributed them for comments and modifications within the scientific committee of the study (JLF, ML, FD, MC, RS, FB, EJ). Thereafter, the survey was sent to a test group of 15 physicians to test both its feasibility and overall quality.

The demographic characteristics of the physicians were collected. The reasons why the physicians perform CRT and how they consider and interpret it were explored. The approach used to perform CRT in routine clinical practice was also assessed and the potential leads to improve clinical practice when using CRT were examined.

### Statistical analysis

Categorical variables were expressed as frequencies and percentages, and continuous variables as mean ± standard deviation. Chi-squared tests were used to perform between-group comparisons. All tests were two-sided, and a *p*-value < 0.05 was considered statistically significant. The descriptive and analytical statistical analyses were computed using the Free Software Foundation’s CRAN R, R version 4.0.4.

## Results

### Test feasibility and quality

The 15 physicians who initially answered the survey test reported no issues in terms of feasibility, and the overall quality was rated as good. The comments were used to refine the final version of the survey.

### Characteristics of the respondents

Among the 6071 physicians who received and opened the email in September and October, 549 (9%) clicked on the survey link. The survey was then completed by 418 (7%) practitioners. The mean ± SD age of the respondents was 39 ± 10 years and the mean ± SD time of clinical experience and practice was 11 ± 9 years. Overall, 60% of respondents worked in an adult intensive care unit (ICU), 30% in a pediatric ICU, and 7% worked in an adult or pediatric emergency department. The respondents practiced mainly in tertiary teaching hospitals (63%), public hospitals (27%), and private hospitals (9%). Among all respondents, 55% worked in units with 11 to 20 beds, and 28% worked in units with more than 20 beds. Overall, 48% of respondents managed 1 to 5 patients with acute circulatory failure per week (Table 1).

**Table 1:** Characteristics of the survey respondents

	Overall	Adult practice	Pediatric practice	*p*-value
n	418	308	110	
Age, years, mean (SD)	39 (10)	39 (11)	40 (8)	0.421
Clinical practice, years, mean (SD)	11 (9)	11 (10)	12 (8)	0.577
**Health care facility location, n (%)**				
Africa	13 (3.1)	13 (4.2)	0 (0.0)	0.029
America	3 (0.7)	1 (0.3)	2 (1.8)	
Other European country	6 (1.4)	5 (1.6)	1 (0.9)	
France	366 (87.6)	272 (88.3)	94 (85.5)	
Overseas France	19 (4.5)	10 (3.2)	9 (8.2)	
No answer	11 (2.6)	7 (2.3)	4 (3.6)	
**Clinical activity, n (%)**				
Adult ICU	251 (60.0)	251 (81.5)	0 (0.0)	< 0.001
Pediatric ICU	127 (30.4)	30 (9.7)	97 (88.2)	
Adult high depency unit	110 (26.3)	108 (35.1)	2 (1.8)	
Pediatric high depency unit	73 (17.5)	12 (3.9)	61 (55.5)	
Adult emergency department	14 (3.3)	14 (4.5)	0 (0.0)	
Pediatric emergency department	14 (3.3)	2 (0.6)	12 (10.9)	
Other	57 (13.6)	40 (13.0)	17 (14.7)	
**Medical specialty, n (%)**				
Intensivist and anesthesiologist	286 (68.4)	283 (91.9)	3 (2.7)	< 0.001
Intensivist	39 (9.3)	27 (8.8)	12 (10.9)	
Cardiologist or pneumologist	4 (0.9)	3 (0.9)	1 (0.9)	
Pediatrician (%)	110 (26.3)	0 (0.0)	110 (100.0)	
Other (%)	29 (6.9)	20 (6.5)	9 (8.2)	
**Health care facility, n (%)**				
Tertiary teaching hospital	265 (63.4)	178 (57.8)	87 (79.1)	< 0.001
Private hospital	39 (9.3)	39 (12.7)	0 (0.0)	
Public hospital	114 (27.3)	91 (29.5)	23 (20.9)	
**Number of beds in health care facility, n (%)**				
0 to10	70 (16.7)	50 (16.2)	20 (18.2)	0.833
11 to 20	230 (55.0)	172 (55.8)	58 (52.7)	
> 20	118 (28.2)	86 (27.9)	32 (29.1)	
**Weekly number of patients with acute circulatory failure in respondent’s care, n (%)**				
< 1 patient a week	81 (19.4)	48 (15.6)	33 (30.0)	< 0.001
1 to 5 patients a week	199 (47.6)	132 (42.9)	67 (60.9)	
5 to 10 patients a week	98 (23.4)	88 (28.6)	10 (9.1)	
> 10 patients a week	40 (9.6)	40 (13.0)	0 (0.0)	

### How physicians consider and interpret CRT

**Fig. 1:**
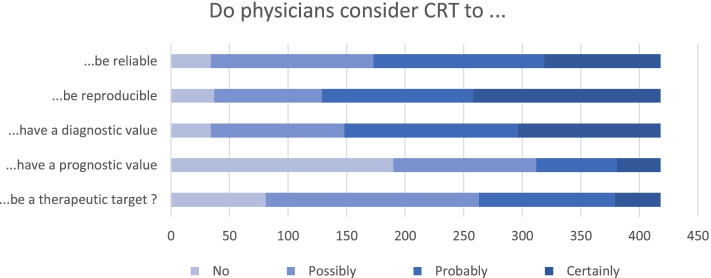
Main considerations regarding CRT according to the 418 survey respondents. CRT: Capillary refill time

Among all respondents, 82% reported using CRT in routine clinical practice, while others did not consider CRT to be reliable (8%) or reproducible (9%) enough for daily use. Furthermore, 308(74%) respondents declared that 3 seconds was the threshold to define abnormal CRT, 29% were convinced CRT could be used to diagnose acute circulatory failure, and 35% thought it could probably have a diagnostic value (Table 2 and Fig. 1). Conversely, 8% considered that CRT had no diagnostic value and 4% felt it was useless in routine clinical practice. Regarding the prognostic value of CRT, 45% thought it had none, while 55% felt CRT probably or certainly reflects tissue perfusion and 47% thought it does not reflect cardiac output. In the setting of acute coronary failure, 37% of respondents considered that a perfusion goal-directed therapy based on CRT measurement could probably or certainly improve patient outcome. In line with these results, 52% of respondents reported never targeting the normalization of CRT during their resuscitation strategy compared to 6% who always do. Finally, 3% declared that a perfusion-targeted protocol based on CRT was in place in their institution to resuscitate patients with acute circulatory failure.

**Table 2:** How respondents use and interpret capillary refill time

	Overall	Adult practice	Pediatric practice	***p***-value
	*n* = 418	*n* = 308	*n* = 110	
**Would you say that capillary refill time is a reliable measurement in clinical practice, n (%)**				
No	34 (8.1)	31 (10.1)	3 (2.7)	< 0.001
Possibly	139 (33.3)	123 (39.9)	16 (14.5)	
Probably	146 (34.9)	96 (31.2)	50 (45.5)	
Certainly	99 (23.7)	58 (18.8)	41 (37.3)	
**Would you say that capillary refill time is a reproducible measurement in clinical practice, n (%)**				
No	37 (8.9)	32 (10.4)	5 (4.5)	0.001
Possibly	92 (22.0)	79 (25.6)	13 (11.8)	
Probably	129 (30.9)	93 (30.2)	36 (32.7)	
Certainly	160 (38.3)	104 (33.8)	56 (50.9)	
**According to you, what is the pathological threshold of capillary refill time, n (%)**				
It depends on the clinical context; we cannot define a threshold	39 (9.3)	34 (11.0)	5 (4.5)	0.002
More than 2 seconds	27 (6.5)	13 (4.2)	14 (12.7)	
More than 3 seconds	308 (73.7)	223 (72.4)	85 (77.3)	
More than 5 seconds	43 (10.3)	37 (12.0)	6 (5.5)	
More than 7 seconds	1 (0.2)	1 (0.3)	0 (0.0)	
**Do you think that capillary refill time can be used to diagnose acute circulatory failure, n (%)**				
No	34 (8.1)	27 (8.8)	7 (6.4)	0.012
Possibly	114 (27.3)	96 (31.2)	18 (16.4)	
Probably	148 (35.4)	103 (33.4)	45 (40.9)	
Certainly	122 (29.2)	82 (26.6)	40 (36.4)	
**Do you think that capillary refill time has a prognostic value in patients with acute circulatory failure, n (%)**				
No	190 (45.5)	122 (39.6)	68 (61.8)	0.001
Possibly	122 (29.2)	97 (31.5)	25 (22.7)	
Probably	69 (16.5)	58 (18.8)	11 (10.0)	
Certainly	37 (8.9)	31 (10.1)	6 (5.5)	
**Do you think that capillary refill time is a reliable surrogate marker of tissue perfusion, n (%)**				
No	54 (12.9)	45 (14.6)	9 (8.2)	0.206
Possibly	135 (32.3)	102 (33.1)	33 (30.0)	
Probably	162 (38.8)	112 (36.4)	50 (45.5)	
Certainly	67 (16.0)	49 (15.9)	18 (16.4)	
**Do you think that capillary refill time is a surrogate marker of cardiac output, n (%)**				
No	195 (46.7)	154 (50.0)	41 (37.3)	0.133
Possibly	150 (35.9)	105 (34.1)	45 (40.9)	
Probably	50 (12.0)	33 (10.7)	17 (15.5)	
Certainly	23 (5.5)	16 (5.2)	7 (6.4)	
**Do you think that a perfusion goal-directed therapy based on capillary refill time could reduce mortality in patients with acute circulatory failure, n (%)**				
No	81 (19.4)	63 (20.5)	18 (16.4)	0.027
Possibly	182 (43.5)	143 (46.4)	39 (35.5)	
Probably	116 (27.8)	79 (25.6)	37 (33.6)	
Certainly	39 (9.3)	23 (7.5)	16 (14.5)	
**Do you think that capillary refill time is useful in clinical practice, n (%)**				
No	18 (4.3)	16 (5.2)	2 (1.8)	< 0.001
Possibly	88 (21.1)	79 (25.6)	9 (8.2)	
Probably	130 (31.1)	100 (32.5)	30 (27.3)	
Certainly	182 (43.5)	113 (36.7)	69 (62.7)	
**Do you follow a resuscitation strategy aiming at normalizing capillary refill time, n (%)**				
Never	218 (52.2)	184 (59.7)	34 (30.9)	< 0.001
Sometimes	175 (41.9)	116 (37.7)	59 (53.6)	
Always	25 (6.0)	8 (2.6)	17 (15.5)	
**Is there a capillary refill time goal directed therapy-based protocol in your institution, n (%)**				
No	375 (89.7)	282 (91.6)	93 (84.5)	0.093
Yes	13 (3.1)	7 (2.3)	6 (5.5)	
I don’t know	30 (7.2)	19 (6.2)	11 (10.0)	
**Do you report capillary refill time in the medical record (%)**				
Never	113 (27.0)	109 (35.4)	4 (3.6)	< 0.001
Sometimes	226 (54.1)	174 (56.5)	52 (47.3)	
Always	79 (18.9)	25 (8.1)	54 (49.1)	
**Do you personally perform capillary refill time?**				
Yes	342 (81.8)	233 (75.6)	109 (99.1)	< 0.001

### How CRT is performed in clinical practice

When performing CRT in patients with acute circulatory failure, 42% of respondents declared assessing CRT 3 to 6 times per day and 50% reported doing it 1 to 2 times per day. CRT is measured at the thorax level for 58% of respondents, at the finger level for 51%, and at the knee level for 40%. To control the pressure applied to the skin, 25% use a specific technique and 75% just apply a firm pressure. The skin is compressed during less than 4 seconds according to 60% of respondents, and 98% assess the compression duration without a chronometer. Forty-six percent averaged at least two consecutive CRT measurements (Table 3).

**Table 3:** Technical details on how CRT is performed in clinical practice

	Overall	Adult practice	Pediatric practice	***p***-value
	*n* = 418	*n* = 308	*n* = 110	
**If you think of the last patient with acute circulatory failure, how often did you assess capillary refill time, n (%)**				
0 /24 h	26 (6.2)	25 (8.1)	1 (0.9)	< 0.001
1 to 2 /24 h	165 (39.5)	141 (45.8)	24 (21.8)	
3 to 6/24 h	137 (32.8)	56 (18.2)	81 (73.6)	
No answer	90 (21.5)	86 (27.9)	4 (3.6)	
**On what part of the body do you assess capillary refill time, n (%)**				
On the fingertip (%)	169 (40.4)	138 (44.8)	31 (28.2)	< 0.001
On the thorax (%)	191 (45.7)	86 (27.9)	105 (95.5)	
On the knee (%)	131 (31.3)	107 (34.7)	24 (21.8)	
On the gingiva (%)	5 (1.2)	4 (1.3)	1 (0.9)	
**How do you control the pressure during the compression of the skin, n (%)**				
You apply a firm pressure	247 (59.1)	164 (53.2)	83 (75.5)	< 0.001
You use the blanching of your finger nail to assess the pressure	74 (17.7)	51 (16.6)	23 (20.9)	
You use a glass microscope slide to assess the blanching of the patient’s skin	7 (1.7)	7 (2.3)	0 (0.0)	
No answer	90 (21.5)	86 (27.9)	4 (3.6)	
**How long does the compression last, n (%)**				
0 to 3 seconds	192 (45.9)	145 (47.1)	47 (42.7)	< 0.001
4 to 7 seconds	119 (28.5)	62 (20.1)	57 (51.8)	
7 to 10 seconds	12 (2.9)	10 (3.2)	2 (1.8)	
> 10 seconds	5 (1.2)	5 (1.6)	0 (0.0)	
No answer	90 (21.5)	86 (27.9)	4 (3.6)	
**How do you measure the duration of the compression, n (%)**				
Using a chronometer	8 (1.9)	8 (2.6)	0 (0.0)	< 0.001
Counting in your head	320 (76.6)	214 (69.5)	106 (96.4)	
No answer	90 (21.5)	86 (27.9)	4 (3.6)	
**How do you measure capillary refill time, n (%)**				
Using a chronometer	12 (2.9)	12 (3.9)	0 (0.0)	< 0.001
Counting in your head	316 (75.6)	210 (68.2)	106 (96.4)	
No answer	90 (21.5)	86 (27.9)	4 (3.6)	
**To evaluate the capillary refill time of a patient at a given moment, how many CRT do you average, n (%)**				
1 measurement	102 (24.4)	81 (26.3)	21 (19.1)	< 0.001
2 measurement	147 (35.2)	99 (32.1)	48 (43.6)	
3 measurement	47 (11.2)	28 (9.1)	19 (17.3)	
the longest of the measurements performed	32 (7.7)	14 (4.5)	18 (16.4)	
No answer	90 (21.5)	86 (27.9)	4 (3.6)	

### Leads to improve clinical practice

**Fig. 2:**
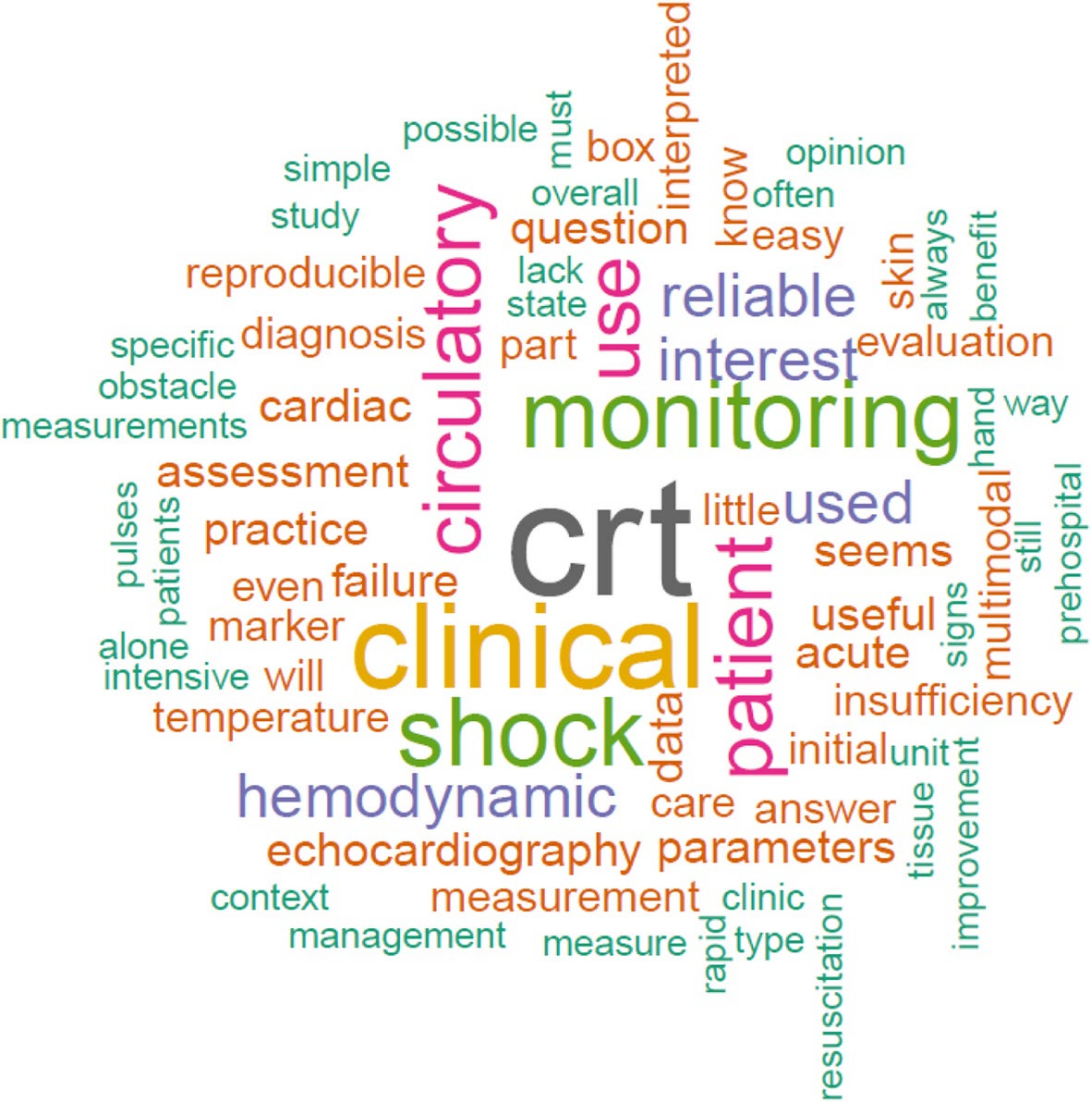
Word cloud obtained from the responses to the open question “Do you have a comment to make about CRT?”

Among the obstacles that limit the widespread use of CRT during routine practice, 51% of respondents pointed out the difficulty in obtaining a reliable measurement, and 42 and 57% reported the lack of medical staff and non-medical staff training, respectively (Table 4). A large majority (90%) considered that CRT measurements could be performed by the non-medical staff. The main leads for improving the daily clinical use of CRT were to delegate measurements to a specifically trained non-medical staff (64%) and to use a dedicated device (54%). In the last open question, which aimed to collect further comments, respondents insisted on the fact that CRT could not be taken as a standalone variable, but should rather be integrated in a multivariable approach (Fig. 2).

**Table 4:** Leads to improve capillary refill time in clinical practice

**What are according to you the obstacles for a more widespread use of capillary refill time, n (%)**				
The lack of clinical benefit	78 (18.7)	67 (21.8)	11 (10.0)	0.004
The difficulty to obtain a reliable measurement	197 (47.1)	146 (47.4)	51 (46.4)	
The fact that obtaining a reliable measurement is time consuming	18 (4.3)	16 (5.2)	2 (1.8)	
The lack of training of non-medical staff	220 (52.6)	154 (50.0)	66 (60.0)	
The lack of training of medical staff	161 (38.5)	134 (43.5)	27 (24.5)	
**According to you, can non-medical staff, following a dedicated training, handle the measurement of capillary refill time, n (%).**				
No	9 2.2)	8 (2.6)	1 (0.9)	0.148
Yes	375 (89.7)	271 (88.0)	104 (94.5)	
No answer	34 (8.1)	29 (9.4)	5 (4.5)	
**What could, according to you, improve the reliability of capillary refill time in clinical practice, n (%)e**				
We should abandon this useless measurement	20 (4.8)	20 (6.5)	0 (0.0)	0.011
CRT should be performed by a trained medical staff	116 (27.8)	89 (28.9)	27 (24.5)	
CRT should be performed by a trained non-medical staff	245 (58.6)	165 (53.6)	80 (72.7)	
CRT should be performed by non-medical staff without specific training	21 (5.0)	17 (5.5)	4 (3.6)	
A specific device that measures CRT should be used	208 (49.8)	155 (50.3)	53 (48.2)

### Differences between adult and pediatric physicians

In routine clinical practice, CRT is used more often by pediatric intensivists (99%) compared to adult ones (76%). The former more frequently reported that CRT is a reliable and reproducible measure and were more confident in the diagnostic value of CRT in the setting of acute circulatory failure. They also more strongly believed in the effectiveness of CRT-based perfusion goal-directed therapy, 63% of them being convinced that CRT is useful at the bedside compared to 37% of adult intensivists. In terms of the technical approach used for CRT measurement, pediatric intensivists used longer compression times and more often averaged multiple measurements (Tables 2 and 3).

## Discussion

This survey found that the majority of physicians use CRT, more likely for its diagnostic than its prognostic value. Less than half of them think that a CRT-based perfusion goal-directed therapy can reduce mortality during acute circulatory failure. The standardized approach used in clinical studies does not seem to be applied during routine practice, as almost none of the respondents reported using a chronometer to measure CRT. Pediatric intensivists were more trustful of CRT in terms of reliability, reproducibility, and diagnostic ability, and their approach was closer to the required standards when compared to adult intensivists.

CRT has been correlated to microcirculatory and perfusion variables such as NIRS [8], video microscopy, and lactate, and has been shown to be a strong prognostic factor in different settings [9–11], including acute circulatory failure [1]. Moreover, CRT has the advantage of being faster, cheaper, and easier to use than specific devices assessing microcirculation and has been used as a triage method [12]. Furthermore, clinical trials suggest a benefit of a CRT-targeted treatment strategy in acute circulatory failure [4, 13, 14] or during the deresuscitation phase [15, 16]. However, the results of the trial by Hernandez et al. [4]were strongly debated and led to ancillary Bayesian analyses [5] which confirmed the potential benefit of such strategies targeting CRT [17]. Ongoing studies may confirm the encouraging results in coming years [18, 19]. Although most of the respondents herein recognized the clinical usefulness of CRT they did not trust its prognostic value and most of them were not convinced that CRT-based perfusion-goal directed therapy could improve the outcome of patients with acute circulatory failure. In the pediatric setting, although the prognostic value of CRT is clear [20] there is less data from interventional studies concerning CRT-targeted therapy than in the adult setting. Nevertheless, the pediatric respondents of the present survey were more confident in the ability to improve outcome if CRT-targeted therapy is applied. This could be due to the fact that CRT-related literature is less recent and more abundant in the pediatric than the adult setting. Moreover, in textbooks considering perfusion assessment in children, CRT is described as one of the signs of circulatory failure and pediatric septic shock [21] . CRT is also used to define septic shock in pediatric randomized clinical trial [22]. This could also be explained by the fact that the pediatric approach is more focalized on noninvasive assessments of circulation as well as the higher need to spare blood in children. For instance, recent guidelines recommend titrating fluid load based on clinical surrogates of cardiac output, which include CRT [23].

In the Hernandez et al. clinical trial, CRT was measured by applying firm pressure to the ventral surface of distal phalanx of the right index finger with a glass microscope slide. Compression time was 10 seconds and the time for normal skin color to return was registered using a chronometer [4]. In the study by Ait-oufella et al. [1], the compression time was 15 seconds and the pressure applied “was just enough to remove the blood at the tip of the physician’s nail illustrated by appearance of a thin white distal crescent (blanching) under the nail”. A chronometer was also used and two measurements were averaged [1]. Others have shown that a syringe filled with air can also be used as a piston to control the pressure applied [24, 25].

Although the absence of link between cardiac output and CRT has been shown [26], its link to tissue perfusion is more obvious [12, 27] and consistent with the loss of hemodynamic coherence in septic shock and acute circulatory failure [28, 29]. Respondents appeared in accordance with these data as they trusted more CRT as a perfusion surrogate than a cardiac output surrogate marker.

The present study has some limitations. With only a 7% response rate, one could argue the results are not representative of the French intensivist population. This rate is however comparable to other studies using the SFAR [30, 31]or the GFRUP networks [32]. Nevertheless, it cannot be excluded that the physicians who decided not to participate were more likely to not use CRT regularly resulting in a possible overestimation of CRT use. This could be due to the networks used to dispatch the survey, as among the anesthesiologist and intensivist members of the SFAR network, only a minority work in ICUs, although the latter are the most likely to manage patients with acute circulatory failure. Given that in France there are 775 anesthesiologists and intensivists working in adult ICUs and 214 of these answered the survey, this represents a 28% response rate from those most likely to manage acute circulatory failure. As the survey was written in French language, this likely impedes the generalizability of the results, as does the fact that a majority of respondents worked in public hospitals. Finally, a selection bias was presumably present, as intensivists with a special interest in CRT were more likely to respond.

Nevertheless, in the absence of a previous evaluation of CRT use in routine practice, these results provide a relevant picture of current practices in France.

## Conclusion

CRT is widely used by physicians managing acute circulatory failure, but most often in a sub-optimal way. Moreover, a gap remains between available data and the degree of confidence in CRT to predict patient outcome or to help clinical decision-making and resuscitation strategies. This may lead to a misunderstanding of the reliability and clinical usefulness of CRT. A simple way to increase the reliability of CRT would be the use of a chronometer, reducing the variation in compression level, and averaging several measurements. Nurse training programs and/or the use of specific devices should help standardize CRT measurements and render CRT-targeted therapy effective in routine clinical practice.

## Supplementary Information

**Additional file 1.** Survey letter.

**Additional file 2.** Questionnaire.

## Data Availability

Data set generated/analyzed during the current study are available from the corresponding author on reasonable request.
