# Supplementary material for: Capillary refill time for the management of acute circulatory failure: a survey among pediatric and adult intensivists
Source: BMC Emerg Med. 2022 Jul 18;22:131. doi: 10.1186/s12873-022-00681-x (PMC9290243; doi:10.1186/s12873-022-00681-x)
Supplement: Supplementary file 1 — Additional file 1. Survey letter. [file 12873_2022_681_MOESM1_ESM.docx]

**Additional files 1: Survey letter**

Dear colleague

We would like you to participate to a survey on capillary refill time in intensive care units.

Capillary refill time measurement is a simple way to assess peripheral perfusion. Recent data suggest the clinical interest of this measurement. Nevertheless, there is no available information on the impact of capillary refill time in clinical practice in the ICU.

This survey will provide to clinician’s information on how capillary refill time is performed in clinical practice and could guide future study in the domain.

Dr Jacquet-Lagrèze
