# Supplementary material for: Capillary refill time for the management of acute circulatory failure: a survey among pediatric and adult intensivists
Source: BMC Emerg Med. 2022 Jul 18;22:131. doi: 10.1186/s12873-022-00681-x (PMC9290243; doi:10.1186/s12873-022-00681-x)
Supplement: Supplementary file 2 — Additional file 2. Questionnaire. [file 12873_2022_681_MOESM2_ESM.docx]

**Additional Files 2 : Questionnaire**

**Question 1:** In which hospital and city do you work?

**Question 2:** In which type of unit do you work? Several answers possible

- Adult Intensive care unit
- Paediatric Intensive care unit
- Polyvalent care
- Paediatric polyvalent care
- Emergencies
- Paediatric emergencies

**Question 3:** How old are you?

**Question 4:** How long (in years) have you been practicing?

**Question 5:** What is your specialty ? Several answers possible

- Anesthesiologist
- Intensive Care Medicine
- Cardiology
- Pneumology
- Paediatrics
- Others

**Question 6:** In which type of structure do you work?

- Public hospital
- University hospital / Teaching hospital
- Private hospital

**Question 7:** How many beds does the intensive care unit where you work have?

- 0 to 10
- 11 to 20
- More than 20

**Question 8:** How often do you manage patients with acute circulatory failure?

- Less than one patient a week
- 1 to 5 patients per week
- 5 to 10 patients per week
- More than 10 patients per weeks

**Question 9:** Would you say than CRT measurement is a reliable measure in your medical practice?

- No
- Possibly
- Probably
- Certainly

**Question 10:** Do you think that CRT measurement is easily reproductible method in your medical practice?

- No
- Possibly
- Probably
- Certainly

**Question 11:** In your opinion, at what point can CRT be considered pathological?

- More than 2 seconds
- More than 3 seconds
- More than 5 seconds
- More than 7 seconds
- It depends on the clinical context, we cannot define a standard value

**Question 12:** Do you think CRT is a component of the diagnosis of shock?

- No
- Possibly
- Probably
- Certainly

**Question 13:** Do you think CRT is a prognostic marker for patients in shock?

- No
- Possibly
- Probably
- Certainly

**Question 14:** Do you think that CRT reliably reflects tissue perfusion in patients in shock?

- No
- Possibly
- Probably
- Certainly

**Question 15:** Do you think the CRT reflects the cardiac output of your patient?

- No
- Possibly
- Probably
- Certainly

**Question 16:** Do you think that therapeutic strategy aims at normalizing CRT would improve mortality in patients in shock?

- No
- Possibly
- Probably
- Certainly

**Question 17:** Do you think CRT is useful in daily clinical practice?

- No
- Possibly
- Probably
- Certainly

**Question 18:** Do you follow a resuscitation strategy for acute circulatory failure based on normalization of CRT?

- Always
- Sometimes
- Rarely
- Never

**Question 19:** Is there a resuscitation protocol in your institution based on CRT?

- Yes
- No
- I don’t know.

**Question 20:** Is the CRT recorded in the patient's medical or monitoring record?

- Always
- Sometimes
- Rarely
- Never

**Question 21:** Do you use CRT in your clinical practice ?

- Yes
- No

**Question 22:** For the last patient with acute circulatory failure that you resuscitated, how often did you perform CRT?

- 0 / 24H
- 1-2 / 24h
- 3-6 / 24h
- after each therapeutic intervention (inotropic filling, vasopressor titration, etc ...)

**Question 23:** On which area of the body do you practice CRT?

- On the finger
- On the chest
- On the knee
- On the gums

**Question 24:** How do you apply pressure?

- You use the whitening of your nail, to make the compression of the patient's skin reproducible.
- You use a glass surface (e.g. myelogram slide) to check the bleaching of the patient's skin
- You apply a firm pressure.
- You don’t know

**Question 25:** How long does the pressure you apply last?

- 0 to 3 seconds
- 4 to 7 seconds
- 7 to 10 seconds
- More than 10 seconds

**Question 26:** How do you measure compression time?

- With a stopwatch
- By counting in your head

**Question 27:** How do you measure recoloration time?

- With a stopwatch
- By counting in your head

**Question 28:** To assess a patient's CRT at a given time, how many measures do you use ?

- 1 measure
- The average of 2 measurements
- The average of 3 measurements
- The average of more than 3 measurements
- The longest time of your measurements

**Question 29:** What are the barriers to using CRT in clinical practice? Several answers possible

- Lack of clinical benefit
- The difficulty of obtaining a reliable measurement.
- The time required to make a reliable measurement.
- Lack of training of paramedical staff in its evaluation
- Lack of training of medical staff in its evaluation
- Others

**Question 30:** Can non-medical staff perform this measurement after specific training?

- Yes
- No

**Question 31:** What do you think could be done to improve the use of CRT in clinical practice? Several answers possible.

- Abandonment of this unnecessary measure
- A realization by better trained doctors
- Delegation of the measure to non-medical staff with specific training
- Delegation of the measure to non-medical staff without specific training
- Automated measurement by a dedicated device
- Others

**Question 32:** Do you have a comment to make about the CRT?
